# Effect of Zinc-fertilizer on varietal performance of finger millet (*Eleusine coracana*) and soybean (*Glycine max*) in western Kenya

**DOI:** 10.1016/j.heliyon.2024.e34829

**Published:** 2024-07-18

**Authors:** Victor Ouma Oluoch, Abigael N. Otinga, Ruth Njoroge

**Affiliations:** University of Eldoret, Department of Soil Science, P.O. Box 1125-30100, Eldoret, Kenya

**Keywords:** Crop response, Depleted micronutrients, Micronutrients, Value cost ratio, Zinc-depleted soils, Zn agronomic use efficiency, Zn uptake

## Abstract

Soil zinc deficiency in Sub-Saharan Africa (SSA) is a major contributor to poor crop responses to nitrogen, phosphorus and potassium (N, P and K) fertilizer leading to low economic returns on fertilizer use. Despite being drought-tolerant crops, finger millet (Eleusine coracana) and soybean (*Glycine max*) yields are consistently lower than 1000 kg ha^−1^ in western Kenya. On-farm trials were conducted in Bungoma and Siaya Counties of western Kenya during two subsequent cropping seasons (long & short rains of 2019) to evaluate the responses of two varieties of finger millet (U15 and SEC915) and soybean (SB19 and SB134) to N, P and K after addition of Zn. Zinc was applied at 0, 1.5, and 3 kg ha^−1^ and N, P and K at blanket rates. Results showed that application of Zn fertilizer alongside N, P and K fertilizer significantly (p < 0.05) increased grain yields, grain Zn concentration and grain Zn uptake. The largest Zn agronomic efficiency (AE_Zn_) was realized when Zn was applied at 1.5 kg ha^−1^ for both crops. Application of Zn was profitable (VCR >1) for growing finger millet and soybean in Bungoma and Siaya during long rains and short rains. We recommend applying Zn along with N, P and K fertilizer in Zn-depleted soils to increase finger millet and soybean yields.

## Highlights

• Improved finger millet and soybean grain yields were obtained when applying Zn alongside N, P and K fertilizer.
• Agronomic efficiency in both finger millet and soybean was largest when Zn was applied at a low level.
• Zinc application is required to enrich grain Zn concentration and uptake in Zn-deficient soils for improved nutrition.
• Investing in Zn for growing finger millet and soybean was profitable (VCR >1).

## 1 Introduction

Zinc (Zn) depletion in most tropical soils is widespread [1]. Replenishment of most of the micronutrients, including Zn, in most soil fertility restoration programmes in SSA, is limited [2]. These practices of inadequate and imbalanced nutrient replenishment [3,4] contribute to low nutrient uptake and thus low crop yields. Beyond being sources of food, finger millet (*Eleusine coracana)* and soybean (*Glycine max)* have high economic value, take a short period to mature (3–4 months), and are resilient to moisture stress [5,6]. In addition finger millet is resilient to emerging pests and diseases; characteristics that are important in adapting to climate change shocks. Despite the mentioned benefits, the average yields of these crops remain low. In western Kenya, for instance, the average grain yield of these crops is less than 1000 kg ha^−1^. This is against a potential yield of 3000 kg ha^−1^ for soybean [7] and 4000 kg ha^−1^ for finger millet [8]. Consequently, cases of food insecurity persist in the region [9].

Most studies in Sub-Saharan Africa (SSA) address the most limiting macronutrients such as N, P, and K. They seldom consider micronutrients like Zn, even though Zn deficiency could cause low productivity [10]. Globally, Zn is limiting in over one-third of cultivated soils [11]. In western Kenya, studies have shown that it is one of the most limiting micronutrients [10,12]. In such soils, Zn deficiency is caused by intensive cropping without adequate Zn replenishment, low organic matter content [13], low total Zn [14], and most likely P-induced Zn deficiency which occurs when a high rate of P is applied to Zn deficient soils [15]. In western Kenya, the interaction of Zn with the mineralogy of the soil type, i.e. Acrisols in Bungoma and Ferralsols in Siaya involves a complex interplay of adsorption-desorption processes influenced by soil pH, organic matter, clay minerals, and iron and aluminium oxides [12]. These soils, typically acidic and rich in kaolinite, have low cation exchange capacity, limiting their ability to retain Zn. Iron and aluminium oxides like goethite, hematite, and gibbsite can adsorb Zn, reducing mobility and availability [16]. Organic matter enhances Zn availability through complexation and slow release, whereas low organic matter content, as shown in western Kenya (Table 1), contributes to low accessible Zn [2].

The interaction of Zn and N produces a synergistic effect that improves crop yield and quality. For instance, previous studies have shown that sufficient supply of Zn improved uptake of N and consequently increased grain yield and Zn concentration in wheat [1,17] and rice [18]. Other than ensuring optimal uptake of Zn, a sufficient supply of N improves absorption of other essential nutrients such as P, Fe, magnesium (Mg) and manganese (Mn) [19]. However, P and Zn have been demonstrated to have antagonistic reaction where excessive supply of either P or Zn in the soil induces the deficiency of Zn and P, respectively. This phenomenon is referred to as P induced Zn deficiency or Zn induced P deficiency, respectively [15]. The Zn induced P deficiency is a rare condition since P fertilizers are usually applied in large quantities as opposed to Zn fertilizers [20]. Phosphorus induced Zn deficiency; however, is a common condition that occurs in soils. Another form of P induced Zn deficiency is Zn dilution in plant shoot as a result of growth responses to P [21]. Zinc and K have a positive effect on improving crop production. For instance, sufficient application of K, Zn and S to soybean increases its seed yield and oil content [22]. Also, a sufficient supply of Zn reduces the exudation of solutes, such as K ions by maintaining cell membrane integrity [22]. The optimal supply of Zn increases the uptake of K and under limited Zn supply K uptake from the soil is reduced [1].

Under limited Zn supply in the soil, yield loss of between 20 and 40 % is estimated, with little or no deficiency symptoms noticed [12,23]. Besides reduced yield, harvested grains are characterized by low Zn concentration [1]. Consequently, human nutrition and food security are affected. Studies have reported over 30 % of the global population suffers from inadequate zinc intake [21], which causes related health complications [24]. Micronutrient deficiencies, such as Zn, can significantly impact developing nations like SSA, with poor households being the most vulnerable. In SSA, about 60–70 % of the population suffers from complications from inadequate Zn intake [14]. Common strategies, such as fortifying food products and genetic fortification, are potentially effective in addressing the region's micronutrient malnutrition [25]. However, these methods are still challenged by ongoing debate regarding food safety for human consumption [26].

It has also been demonstrated that foliar Zn fertilizer application efficiently enriches food crop grains with Zn to desirable levels for human nutrition [27]. However, foliar application of Zn is only efficient in shorter period under sufficient rainfall. This implies that the foliar application of Zn may not be sustainable and reliable for places like western Kenya and SSA that receive unreliable rainfall. Adverse effects such as salt injury to crops due to high concentrations of Zn may occur when the crop faces moisture stress. Zinc application through the soil has been reported to increase DTPA-Zn concentration and improve Zn uptake in harvested crops [28]. Therefore, through an on-farm trial for two subsequent cropping seasons (Long & short rains of the year 2019) in both Bungoma and Siaya, the study sought to assess the effects of different Zn fertilizer levels, alongside standard N, P, and K fertilization, on grain yield, Zn agronomic efficiency, grain Zn concentration, grain Zn uptake, and value cost ratio in finger millet and soybean varieties. The study compared these parameters across the two experimental sites, cropping seasons, and crop varieties. As noted by Ref. [29], different varieties can exhibit varying levels of efficiency in utilizing applied nutrients, including Zn, which could lead to differences crop responses. Despite existing research on Zn application, the specific effects of different finger millet and soybean varieties on Zn uptake and yield are not well-documented. The varieties used were U15 and SEC915 for finger millet and SB19 and SB134 for soybean, chosen for their economic value and drought tolerance. These varieties were selected based on their adaptation to local agro-climate conditions and differing genetic potentials for yields and nutrient uptake. By comparing these varieties, the study aimed to determine which specific ones would respond best to Zn fertilization, thereby optimizing Zn application rates. This approach sought to improve overall crop productivity and nutritional quality and provide the best economic returns for farmers in the region. The initial chemical conditions of the study regions, characterized by low organic carbon and Zn deficiency (Table 1), were suitable for testing the hypothesis that Zn application would significantly improve crop performance.

## 2 Materials and methods

### 2.1 Study site

The study was conducted in Bungoma and Siaya Counties of western Kenya during the long and short rains of the year 2019. On-farm trials were set up in Kanduyi village, Bumula Sub County, Bungoma County, positioned between latitudes 0° 28′ and 1° 30′ north and longitudes 34° 20′ and 35° 15′ East [30] and Masat East village, Ugenya Sub County, Siaya County, positioned between latitudes 0° 08′ and 0° 47′ North and longitudes 34°25′ and 34° 38′ east [31]. The altitude ranges from 1200 to 4321 (At Mount Elgon) meters above sea level in Bungoma County [30] and 1300 to 1500 m above sea level in Siaya County [31]. Both sites experience a bimodal rainfall pattern, with the long rains occurring between March and July and the short rains between August and December. In Bungoma, the annual precipitation varies from 1800 to 2000 mm [32] with expected rainfall ranging between 500 and 1000 mm during long rains and 430–800 mm during short rains [33]. In Siaya, annual rainfall ranges between 1450 and 1900 mm [34] with long rains season receiving precipitation of between 750 and 950 mm while short rains receive rainfall of between 600 and 800 mm [35]. The main socioeconomic activity in both regions is mixed farming. The regions largely depend on small-scale mixed farming activities such as crop and livestock production. The farming activities are dominated by crops such as maize, common beans, groundnuts, sorghum, cassava, green grams, peas, sweet potatoes, soybeans, and finger millets. Acrisols dominate Bungoma while Ferralsols occupy most parts of Siaya. Despite the low soil fertility experienced in both regions, they occupy the largest areas where finger millet [8] and soybean [7] are produced in western Kenya.

The site selection was based on the farmer's observations that included crop responses to previous fertilizers applied, trends of yields obtained, and the farms' management history, such as the type of fertilizer used and management of soil acidity. With the help of farmers, secondary data, soil test results and farmer willingness to host the experiments on their farm, we selected farms that had a slope of <5 % to reduce the effect of soil fertility gradient [2] with no history of liming within the past five years so as not to compromise Zn solubility [15]. Before planting, random core samples of soil (0–20 cm) were collected from the experimental sites for laboratory analysis of selected physical and chemical properties. These were carried out following the standard procedures outlined by Ref. [36].

Before the setting up of the experiment, soil samples were collected from each plot for site characterization (presented in section 2.2). Nine core samples were randomly collected from a plough layer of 0–20 cm in each plot using a soil auger. The collected random samples were mixed thoroughly to obtain a composite sample for each plot. Analysis of soil samples was done at the University of Eldoret Soil Science Laboratory following the procedures described by Ref. [36]. The composite soil samples of about 500 g were air-dried in the greenhouse at the University of Eldoret and then passed through 2 mm and 0.25 mm sieves. A sub-sample was taken from each sample for analyses of particle size (Hydrometer method), pH (water), total N (colorimetric method), available P (Olsen's method), available K (1 M NH_4_OAc extraction method), total C (Walkey and Black method), and available Zn (EDTA extraction method). Further, dry grains (12–13 % moisture content) were sampled after every harvesting period and ground into a fine powder before analysis for Zn as per the laboratory manual by Ref. [36].The initial soil characteristics of the experimental sites are presented in Table 1. Soils from both sites were moderately acidic with a pH of 5.34 and 5.68 for Siaya and Bungoma, respectively. The level of total N was low in Bungoma (0.23 %) and moderate in Siaya (0.29 %), while that of available P was low (<10 mg kg^−1^) in both sites. The Exchangeable K level was low (<175 mg kg^−1^) in Bungoma with a value of 69.6 mg kg^−1^, while that in Siaya was high at 321.43 mg kg^−1^. Zinc levels (<10 mg kg^−1^) and total carbon (<3 %) were low in both sites. The textural class of the soils was classified as sandy clay loam in Bungoma and clay loam in Siaya. These results show that Zn, N, P and C were limiting crop production across the sites, thus requiring adequate application of the respective fertilizer inputs.

### 2.2 Treatments and experimental design

The fertilizer treatments comprised three levels of Zn as 0, 1.5, and 3 kg ha^−1^,designated as Zn1, Zn2 and Zn3 al, The criteria for using theses Zn doses were primarily based on previous research by Ref. [2] and recommendations from the National Accelerated Agricultural Input Access Programme (NAAIAP) and the Kenya Agricultural Research Institute (KARI), which suggest applying 3 kg ha^−1^ Zn in the region [12]. [2] offered valuable insights into the effects of Zn fertilization on maize yields. However, our study addresses a gap identified in Ref. [2] by focusing on different crops, finger millet and soybean, and exploring their specific responses to Zn fertilization. This broader approach aimed to provide a more comprehensive understanding of Zn fertilization's impact on diverse crop systems and conditions. Alongside Zn rates, a standard N, P and K fertilizer was applied in blanket at 100 kg ha^−1^ N, 30 kg ha^−1^ P, and 50 kg ha^−1^ K for millet and P & K at 30 kg ha^−1^ P and 50 kg ha^−1^ K for soybean. A fourth treatment was also included that was absolute control (without Zn and NPK). The same fertilizer Zn rates were applied for both crops during long and short rains. Nitrogen was not applied to soybean based on the principle that the crop fixes enough nitrogen through the process of biological nitrogen fixation [37].

Nitrogen (N) was supplied as urea, P was sourced from superphosphate while muriate of potash (MOP) supplied K (Table 2). The experimental design used was a factorial arrangement fitted in a Randomized Complete Block Design (RCBD) with three replicates. The two early maturing, drought-tolerant, pest and disease-tolerant seed varieties of finger millet-U15 and SEC915- were obtained from International Crops Research Institute for the Semi-Arid Tropics (ICRISAT) through EASTCOM Foods (a seed distributor in Siaya), while soybean varieties -SB19 and SB134- were obtained from International Institute for Tropical Agriculture (IITA) Maseno-Kenya.

### 2.3 Trial management

The experimental plots were maintained throughout the research period by following all the recommended agronomic practices and protected from livestock grazing. At the beginning of each cropping season, the plots, each measuring 4 m by 5.5 m, were prepared to fine-tilth using conventional hand hoes and boundaries from each plot marked with pegs. Furrows were dug in each plot based on crop spacing. Each finger millet plot had 15 furrows with an inter-row spacing of 30 cm and intra-row spacing of 5 cm, while each soybean plot had ten furrows with an inter-row spacing of 45 cm and intra-row spacing of 5 cm. This gave a plant population of 668,000 per hectare for finger millet and 446,000 per hectare for soybeans. Fertilizer treatments were banded within the furrows in the respective plots except for the control. A thin layer of soil was used to cover the fertilizers before seed placement with all being applied once except N which was applied in two equal splits. The first split was applied at planting, while the second was applied six weeks after finger millet emergence. Two seeds of each crop were sown per hill and later, after emergence, thinned to one plant.

Weeding was carried out twice using conventional hand hoes during each cropping season to ensure weed-free plots. The first weeding was done three weeks after crop emergence, while the second weeding was performed again after three weeks. Pests and fungal diseases were controlled using effective pesticides and fungicides. Harvesting of all plots was done at physiological maturity in the fourth month after crop emergence, and dry yield was determined.

### 2.4 Data collection

#### 2.4.1 Grain yield determination

An effective area measuring 3 m by 3 m in each plot was harvested, leaving 0.75 m and 1 m away from width and length edges, respectively. Total and sub-sample fresh weights recorded from both finger millet heads and soybean pods per effective net area. Sub-samples of the harvestable part of the crops taken to the University of Eldoret for drying. Dry heads and pods from the sub-samples were weighed before threshing. After threshing, grains were separated from stover, and their dry weight recorded. Grain yields were expressed in kilograms per hectare (kg ha^−1^) after adjusting for grain moisture to 13 %. Grain yield was calculated by taking the total sample fresh weight, multiplying it by the sub-sample dry weight, and then dividing this product by the sub-sample fresh weight. The resulting value was then multiplied by 10000 square meters and divided by nine square meters to covert the yield to kg ha.^−1^

#### 2.4.2 Zinc agronomic efficiency (AE_Zn_)

The AE_Zn_ was determined by calculating the difference between grain yield with Zn application and that without. The result obtained was divided by the amount of Zn fertilizer applied as indicated by Eq (1).

Eq1 $${\text{AE}}_{\text{Zn}\;}\left(\;\left(kg\;grain\right)/\left(kg\;Zn\right)\right)=\left(\frac{grain\;yield\;with\;Zn\;application-grain\;without\;Zn\;application}{\;amount\;of\;Zn\;applied}\;\right)$$

where;

AEZn ((kg grain)/(kg Zn)): Agronomic Efficiency of Zinc, expressed as the increase in grain yield per unit of Zn applied.

Grain yield with Zn application (kg): The yield of grain obtained from plots where Zn was applied.

Grain yield without Zn application (kg): The yield of grain obtained in plots without Zn application (Zn1).

Amount of Zn applied (kg): The total amount of Zn applied.

#### 2.4.3 Grain zinc uptake

Dry grains (12–13 % moisture content) from each treatment were ground into a fine powder before analysis for Zn concentration according to the procedures documented in Ref. [36]. The amount of Zn uptake was calculated and expressed in kilograms per hectare as indicated in Eq (2).

Eq 2 $$Zinc\;uptake\;\left(kg\;ha{‾}^{\;1}\right)=\left(\frac{Zn\;content\;\left(mg/kg\;\right)}{\;1000000}\;\right)\times yield\;\left(kg\;ha{‾}^{\;1}\;\right)$$

where;

Zn uptake (kg ha^−1^) is the total Zn uptake in grains per hectare, Zn content (mg kg^−1^) is the concentration of Zn in the grains and,

Yield (kg ha^−1^) is the grain yield per hectare.

#### 2.4.4 VCR (partial budget) analysis

Economics of each fertilizer treatment for finger millet and soybean was calculated using Value Cost Ratio (VCR) analysis as described by Ref. [2]. An average of 100 Kenya shillings (KES) to one USD dollar ($) accounted for inflation effects. The market price per kilogram of fertilizer was $ 0.56 (urea), $0.7 (TSP), $ 0.84 (MOP), and $ 12 (ZnO). The market price for grain yield in 2019 was $ 1.5 and $ 1 for a kilogram of finger millet and soybean, respectively.

VCR e values obtained were calculated using Eq (3).

Eq3 $$\text{VCR}=\left(\frac{etra\;yiled\;obtained\;due\;to\;fertilizer\;use\;\left(\frac{kg}{ha}\right)*price\;of\;grain\;\left(\frac{\$}{kg}\right)}{\;amount\;of\;fertilizer\;applied\;\left(\frac{kg}{ha}\right)*cost\;of\;fertilizer\;\left(\frac{\$}{kg}\right)}\right)$$

A VCR value greater than one (>1) means a net profit, while a value less than one (<1) denotes a net loss as long as other production costs remain unchanged due to fertilizer application. It implies that having a larger VCR translates to a greater return on the particular fertilizer. Usually, a VCR value of ≥2 is considered a critical threshold for assessing the profitability of fertilizer use [2,38,39]. In other words, for every amount spent on fertilizer, at least double the amount invested should be obtained from additional crop yield for that particular fertilizer use to be financially viable.

### 2.5 Statistical analysis

The data collected from the field was managed using Microsoft Excel, and data were subjected to analysis of variance (ANOVA) using Genstat Statistical package (14th edition) to determine the effects of treatments. Normality of the residuals was assessed using normal probability plots. Homogeneity of variances was also checked using residual plots. Fertilizer treatments and variety were considered fixed factors, while random factors included season and site. Effects of fertilizer treatments, variety, season, and site, as well as their interactions, were evaluated. The standard error of differences (SED) between treatments was calculated at a 5 % level of significance. Descriptive analyses were performed and results were presented in bar charts with error bars and box and whisker plots.

## 3 Results

### 3.1 Effect of Zn fertilizer on finger millet and soybean grain yields

Table 3 shows the grain yield of finger millet as affected by the Zn fertilizer application. Zinc fertilizer significantly (p < 0.05) increased the grain yields of finger millet. Application of Zn at the highest level (3 kg ha^−1^ Zn) gave significantly larger grain yields compared to the other treatments. The least grain yield was observed in plots that did not receive fertilizer. Both varieties of finger millet showed a similar trend in performance in both seasons, with long rains recording a larger yield of 1910 kg ha^−1^ than short rains (1020 kg ha^−1^). In Siaya, larger yields were obtained in the short rains. On average, larger yields were observed in Bungoma (1780 kg ha^−1^) than in Siaya (1150 kg ha^−1^).

Soybean yields as affected by Zn fertilizer are presented in Table 4. These followed a similar trend as the finger millet, with an application of Zn at the highest rate giving the largest yields (p < 0.05). Variety also had a significant (p < 0.05) effect on soybean grain yield, with SB19 recording larger yield than SB134 both at Zn application of 3 kg ha^−1^ Zn. Soybean grain yields differed with growing seasons. The grain yields were at 1390 kg ha^−1^ during long rains, and they were reduced by 10 % during short rains. At the sites, long rains recorded a larger yield than short rains in Bungoma, while in Siaya; yields in the short rains were larger than the yields in the long rains. Interestingly, in Siaya, both soybean varieties gave larger yields during the short rains compared to long rains. Fertilizer * variety interaction was also significant (P < 0.05) on soybean grain yield. The season* site, as well as fertilizer*season*site interactions, were highly significant (P < 0.05) on soybean grain yield.

### 3.2 Effect of Zn fertilizer on agronomic efficiency of Zn in finger millet and soybean

Zinc fertilizer application significantly improved the AE_Zn_ (p < 0.05) in finger millet (Table 5) and soybean (Table 6). For finger millet, 1.5 kg ha^−1^ Zn resulted in a higher AE_Zn_ (252.17 kg grain kg^−1^ Zn) than 3 kg ha^−1^ Zn (189.34 kg grain kg^−1^ Zn). In Bungoma, AE_Zn_ was 228.19 and 205.73 kg grain kg^−1^ Zn for 1.5 and 3 kg ha^−1^ Zn, respectively. In Siaya, applying 3 kg ha^−1^ Zn gave an AE_Zn_ of 276.14 and 1.5 kg ha^−1^ Zn resulted in 172.96 kg grain kg^−1^ Zn. Varietal differences showed U15 with higher AEZn (248.26 kg grain kg-1 Zn) than SEC915 (193.25 kg grain kg-1 Zn). Similarly, a varietal effect (p < 0.05) was observed in the AE_Zn_, with U15 recording a larger value of 248.26 kg grain kg^−1^ Zn than SEC915, which gave 193.25 kg grain kg^−1^ Zn. Within sites, SEC915 recorded a greater AE_Zn_ of 238.28 kg grain kg^−1^ Zn compared to U15 (195.65 kg grain kg^−1^ Zn) in Bungoma, while in Siaya, U15 gave a larger AE_Zn_ of 300.87 kg grain kg^−1^ Zn than SEC915 (148.23 kg grain kg^−1^ Zn).

For soybean, application of 1.5 kg ha^−1^ Zn gave higher AE_Zn_ (222.05 kg grain kg^−1^ Zn) than 3 kg ha^−1^ Zn (160.23 kg grain kg-1 Zn). At sites, Zn application at 1.5 and 3 kg ha^−1^ gave AE_Zn_ of 216.51 and 144.93 kg grain kg^−1^ Zn, respectively, in Bungoma and 227.59 and 175.52 kg grain kg^−1^ Zn, respectively, in Siaya. Just like in the finger millet, varietal effect (p < 0.05) showed SB19 with higher AE_Zn_ (215.32 kg grain kg^−1^ Zn) than SB134 (166.96 kg grain kg^−1^ Zn). Specifically, SB19 and SB134 recorded AE_Zn_ of 225.17 and 136.28 kg grain kg^−1^ Zn, respectively, in Bungoma and 205.47 and 197.64 kg grain kg^−1^ Zn, respectively in Siaya. Consistently, seasons influenced the AE_Zn_ (p < 0.05) in soybean; with the long rains recording a larger AE_Zn_ of 209.67 kg grain kg^−1^ Zn than the short rains (172.61 kg grain kg^−1^ Zn). Within the sites, the long rains recorded a greater AE_Zn_ of 220.98 kg grain kg^−1^ Zn than short rains, with an AE_Zn_ of 140.47 kg grain kg^−1^ Zn in Bungoma, while in Siaya, short rains recorded a larger AE_Zn_ of 204.74 kg grain kg^−1^ Zn compared to the long rains (198.37 kg grain kg^−1^ Zn).

### 3.3 Effect of Zn fertilizer on grain Zn concentration in finger millet and soybean

**Fig. 1:**
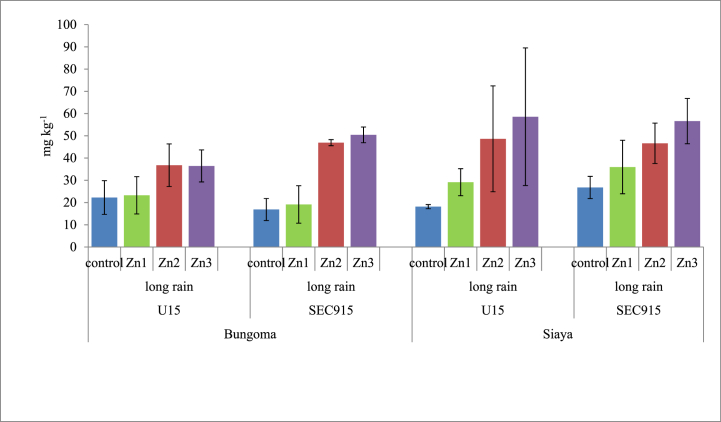
Grain Zn concentration in finger millet as influenced by Zn application in Bungoma and Siaya during LRs season of 2019. Vertical bars stand for standard deviation of mean (Stdv).

**Fig. 2:**
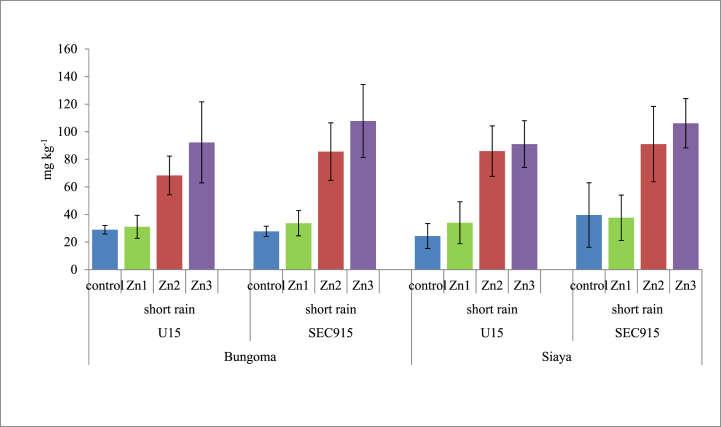
Grain Zn concentration in finger millet as influenced by Zn application in Bungoma and Siaya during SRs season of 2019. Vertical bars stand for standard deviation of mean (Stdv).

As shown in Figs. 1–2, significant (p < 0.05) increases in grain Zn concentration in finger millet were observed with increasing Zn applications irrespective of sites and seasons. Zinc application at 3 kg ha^−1^ gave the largest grain Zn concentration, in both Bungoma and Siaya. In Bungoma, application of Zn at 3 kg ha^−1^ Zn gave the largest grain Zn concentration during long and short rains. In the same region, U15 gave largest grain Zn concentration when Zn was applied at 3 kg ha^−1^ in the long and short rains. In Siaya, a similar trend was observed, with short rains recording greater grain Zn concentration than long rains in both finger millet varieties. Specifically, the SEC915 variety recorded greater values at Zn application of 3 kg ha^−1^ Zn during long and short rains. Improved grain Zn concentration was also observed in the U15 variety, when 3 kg ha^−1^ Zn was applied.

**Fig. 3:**
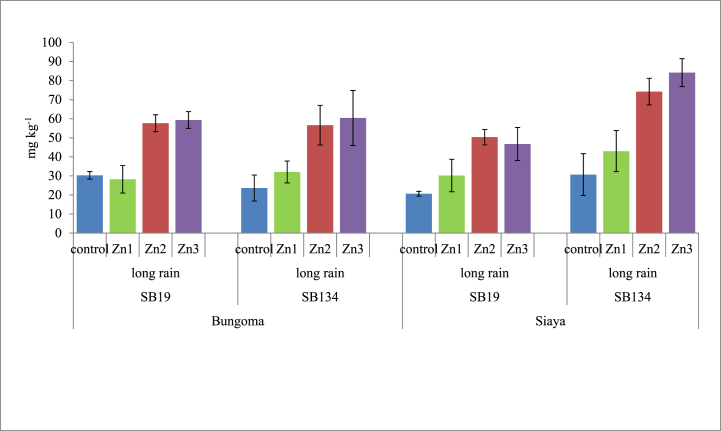
Grain Zn concentration in soybean as influenced by Zn application in Bungoma and Siaya during the LRs season of 2019. Vertical bars stand for standard deviation of mean (Stdv).

**Fig. 4:**
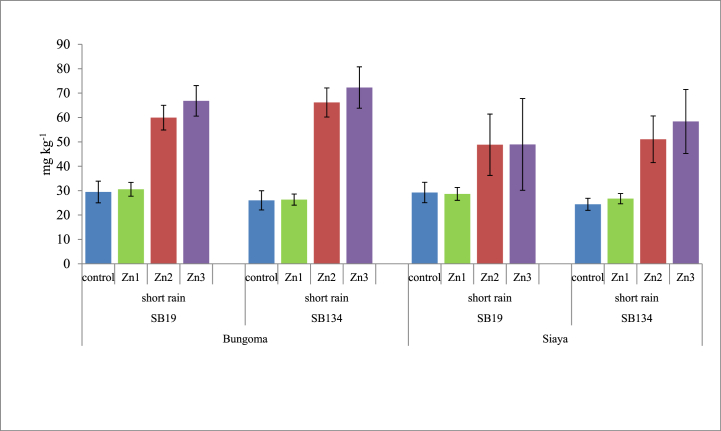
Grain Zn concentration in soybean as influenced by Zn application in Bungoma and Siaya during the SRs season of 2019. Vertical bars stand for standard deviation of mean (Stdv).

Grain Zn concentration in soybean as influenced by Zn application is shown in Figs. 3–4.Like in finger millet, grain Zn concentration was enhanced in soybean with Zn application, significantly increasing grain Zn concentration above the plots that received standard fertilization without Zn. Applying Zn at the highest rate (3 kg ha^−1^) recorded greater grain Zn concentration in both SB19 and SB134 varieties than measured in plots that received standard fertilizer without Zn in the long and short rains in Bungoma. In Siaya, improved grain Zn concentration was observed with Zn application in both soybean varieties. Applying 3 kg ha^−1^ Zn gave greater grain Zn concentration than when 1.5 kg ha^−1^ Zn was applied in SB134 in the long rains and short rains. SB19 variety recorded the highest grain Zn concentration, with 1.5 kg ha^−1^ Zn during long rains and 3 kg ha^−1^ Zn during the short rains.

### 3.4 Effect of Zn fertilizer on grain Zn uptake in finger millet and soybean

**Fig. 5:**
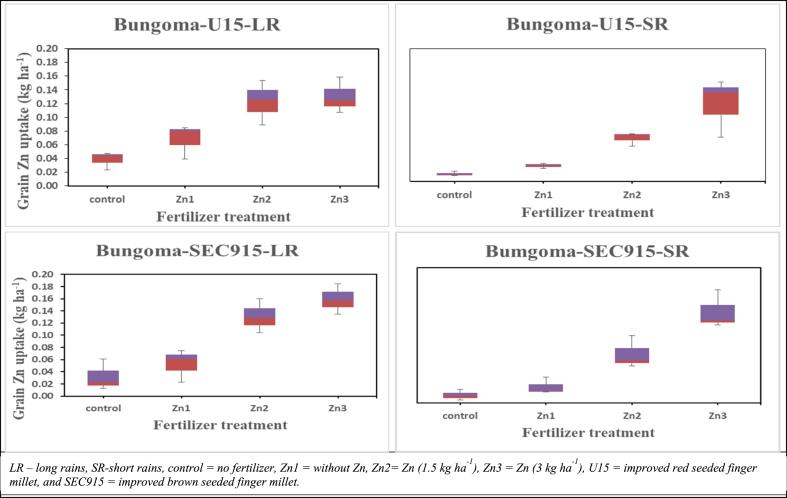
Grain Zn uptake in finger millet as influenced by Zn application in Bungoma during the LRs and SRs seasons of 2019.

**Fig. 6:**
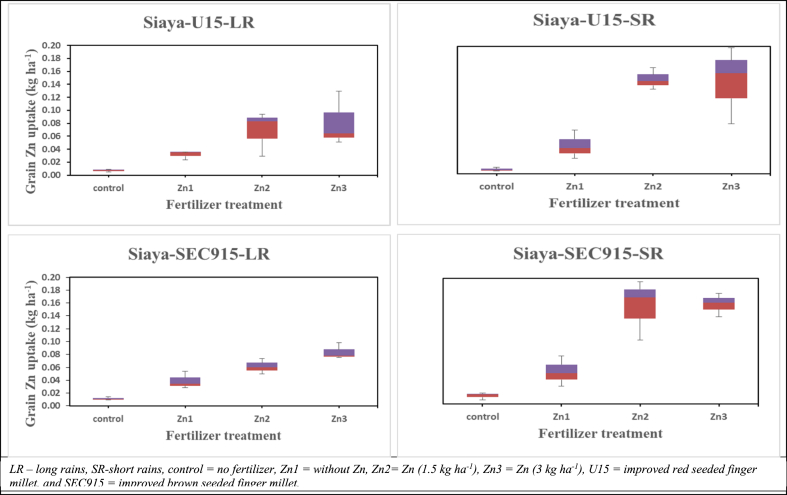
Grain Zn uptake in finger millet as influenced by Zn application in Siaya during the LRs and SRs seasons of 2019.

**Fig. 7:**
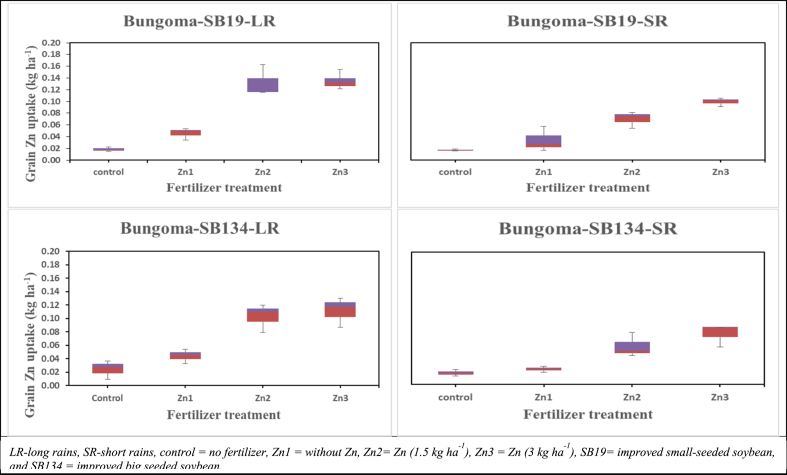
Grain Zn uptake in soybean as influenced by Zn application in Bungoma during the LRs and SRs seasons of 2019.

**Fig. 8:**
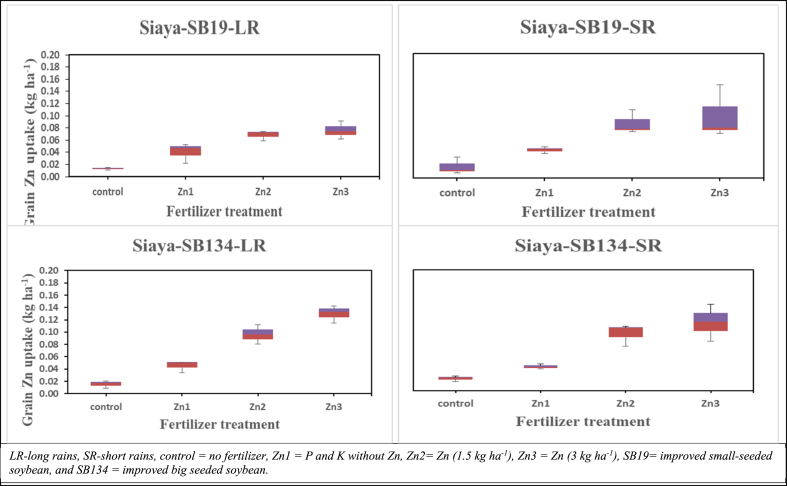
Grain Zn uptake in soybean as influenced by Zn application in Siaya during the LRs and SRs seasons of 2019.

Finger millet (Figs. 5–6) and soybean (Figs. 7–8) showed significant increases in grain Zn uptake with Zn fertilizer application. In Bungoma, finger millet (U15) had a 91 % and 404 % increase with 3 kg ha^−1^ Zn and 80 % and 182 % increase with 1.5 kg ha^−1^ Zn during the long and short rains, respectively. The SEC915 variety had a 200 % and 462 % increase when 3 kg ha^−1^ Zn was applied and a 147 % and 200 % increase with 1.5 kg ha^−1^ Zn in the long and short rains, respectively. In Siaya, U15 had a 161 % and 229 % increase when Zn was applied at 3 kg ha^−1^ and a 119 % and 236 % increase with 1.5 kg ha Zn^−1^ in the long and short rains, respectively. On the other hand, an increase in SEC915 showed a115 % and 211 % increase with 3 kg ha^−1^ Zn and a 56 % and 205 % increase with 1.5 kg ha^−1^ Zn during long and short rains, respectively.

For soybean in Bungoma, SB19 had a 195 % and 194 % increase with 3 kg ha^−1^ Zn and a 184 % and 105 % increase with 1.5 kg ha^−1^ Zn in the long and short rains, respectively. SB134 variety gave 152 % and 230 % increases when 3 kg ha^−1^ Zn was applied and 134 % and 130 % with 1.5 kg ha^−1^ Zn in the long and short rains, respectively. In Siaya, SB19 had a 90 % and 119 % increase when 3 kg ha^−1^ Zn was applied and a 70 % and 91 % increase with 1.5 kg ha^−1^ Zn in the long and short rains, respectively. SB134 recorded a 188 % and 182 % increase with 3 kg ha^−1^ Zn and a 113 % and 135 % increase with 1.5 kg ha^−1^ Zn during the long and short rains, respectively.

### 3.5 Economic benefit of investing Zn fertilizer for finger millet and soybean grain production

Economic benefits of adding Zn fertilizer indicate it is worth investing in Zn for both finger millet and soybean production across the two sites (Table 7, Table 8). On average adding Zn fertilizer while growing finger millet gave significant (p < 0.05) increases in profitability, with a 30 % increase above the average value of VCR of 3.72 observed from the standard agronomic practices, irrespective of variety, sites and seasons. In general growing finger millet with Zn fertilizer irrespective of variety resulted to large economic returns during long rains compared to short rains. This was opposite in Bungoma where larger profits from using Zn fertilizer were observed during short rains irrespective of the varieties grown. Nevertheless, the largest returns on fertilizer investment is observed (average VCR = 7.5) during long rains and when variety U15 is grown irrespective of rate of Zn applied. In Siaya application of Zn at 1.5 kg ha^−1^ resulted to the largest returns of fertilizer investment (VCR = 6.72) when variety U15 was grown during short rains.

A similar trend was also observed in soybean, where Zn application resulted to increased economic benefits of fertilizer use (Table 8). Specifically, investing in Zn at 1.5 kg ha^−1^ in Bungoma was the most profitable investment during long rains irrespective of the varieties grown. In Siaya, applying Zn up to 3 kg ha^−1^ and growing SB19 during short rains was the most profitable fertilizer investment.

## 4 Discussion

### 4.1 Effect of Zn fertilizer on finger millet and soybean grain yields

The benefits of Zn fertilizer application were evident in the considerable improvements in finger millet and soybean grain yields compared to where Zn was not applied. In our study, yields were larger at the highest application rate of Zn (3 kg ha^−1^) across crop varieties, seasons, and sites. This finding corroborates various studies in literature. As demonstrated in our study, applying Zn is essential to address the limitations of nutrient deficiencies, as stipulated by "Liebig's law of the minimum" and [40]. [18,22] also reported increases in cereal grain yield when Zn was applied together with N and K. Similar results have also been reported in legumes. For instance Ref. [41], reported that applying zinc-enriched farmyard manure improved soil available N, organic carbon, extractable Zn, and other nutrients, which consequently increased mung bean grain yield. Further [42], reported improved growth in cereals (maize and wheat) and legumes (chickpea and mung bean) when Zn was applied alongside copper. Similar results have also been reported in SSA. For instance Ref. [40], reported increased maize yield by 25 % over macronutrient application only in SSA when S and limiting micronutrients such as Zn, Cu, Fe, Mo, and B are applied alongside the macronutrients.

Zinc deficiency limits crop yield in the study areas. This micronutrient plays a crucial role in nutrient metabolism, which promotes their uptake and utilization [22] and is also actively involved in increasing the dry matter yield of crops [43]. Additionally, metabolic functional roles played by Zn, such as internode elongation, gene expression, photosynthesis, and protection against diseases [20,21], could have contributed to the improved response of finger millet and soybean to applied fertilizers.

Application of inorganic fertilizer alone is not adequate for increasing crop yield. Both finger millet and soybean could hardly reach their potential yields of 4000 kg ha^−1^ [8] and 3000 kg ha^−1^ [7], respectively. Crop yields are limited when any essential nutrient element is limited despite the sufficient supply of others. For instance, the initial characterization of our study sites revealed soils with low organic carbon (Table 1), an indicator of depleted soil fertility in the region that could have played a significant role in grain yield production. Reduced tillage can help to reduce loss of organic carbon in the study areas.

However, our study also presents new insights specific to the varieties and conditions in western Kenya. The different response of both finger millet varieties and soybean varieties to Zn application highlights the importance of variety selection in optimizing Zn utilization strategies. For instance, the U15 variety of finger millet recorded higher yields compared to SEC915, suggesting a variety-specific response to Zn fertilization that has not been extensively explored in western Kenya. Moreover, our study underscores the significance of seasonal variations and site-specific conditions in influencing, crop response too Zn fertilization. The consistently higher yields during the long rains compared to short rains, particularly in Bungoma, indicate that water availability plays a crucial role in Zn uptake and utilization. This seasonal effect emphasizes the need for tailored fertilizer use strategies for local climate conditions.

The special contributions of our work also include the detailed assessment of soil conditions in the study areas. The initial soil characterization (Table 1) revealed low organic carbon and acidic soils, significantly influencing nutrient availability and crop yield potential. Addressing these underlying soil fertility issues is crucial for effective Zn fertilizer use, as evidenced by our findings. This site-specific analysis adds depth to the broader understanding of Zn's role in enhancing crop productivity under suboptimal soil conditions.

### 4.2 Effect of Zn fertilizer on agronomic efficiency of Zn in finger millet and soybean

Agronomic efficiency of Zn was largest at a lower rate of 1.5 kg ha^−1^ Zn compared to when the rate was doubled. This is consistent with [44], who reported that the agronomic effectiveness of Zn is realized at low application rates. In this context, smaller AE_Zn_ at 3 kg ha^−1^ Zn could be attributed to antagonistic reactions with the nutrients such as P and possibly Fe and Ca, and also to luxury consumption of Zn. Applying either a larger amount of Zn or P fertilizer is reported to induce deficiency of either P or Zn nutrients, respectively [1,21,45]. Zinc fertilizer efficiency is much better when soils are already rich in other nutrients. As mentioned in section 4.1, adequate amounts of organic carbon would significantly improve the efficiency of fertilizer Zn due to benefits of organic C in tropical soils. Our results suggest that realizing optimal AE_Zn_ requires an appropriate Zn and P ratio, warranting further investigation.

A significant (p < 0.05) varietal effect was observed across the two varieties of finger millet (Table 5) and soybean (Table 6). Zinc uptake and its use among various plant species and cultivars are reported to be associated with different absorption mechanisms [29,46]. [47] noted that genetic and physiological factors and environmental interactions control plant growth and nutrient use efficiency. Crops that are effective in absorbing and utilizing nutrients improve the efficiency of applied fertilizers, lowering input costs and preventing nutrient losses. Therefore, identification of traits in crop cultivars such as nutrient absorption, transport, mobilization, and utilization would considerably provide insights into breeding initiatives to improve Zn fertilizer efficiency in finger millet and soybean.

### 4.3 Effect of Zn fertilizer on finger millet and soybean grain Zn concentration and uptake

Our study indicated that grain Zn concentration and Zn uptake were larger when Zn was applied, with the highest application of Zn up to 3 kg ha^−1^ recording greater values across crop varieties, seasons, and sites. These increases in grain Zn concentration and uptake were consistent with various studies in the literature. For instance Ref. [48], reported increased grain Zn concentration in wheat with Zinc sulphate application as either a soil supplement or foliar. Studies on rice and maize also showed a significant increase in grain Zn concentration with Zn fertilizer was applied in Zinc-deficient soils [49,50]. Similar results have been reported in maize and wheat [25,51] and for rice grown in Zn-fertilized soil [52,53].

Similar results have also been observed in legumes. For instance Ref. [54], realized improved grain Zn concentration in chickpea with 10 kg ha^−1^ Zn application in zinc-deficient soils. However, the Zn application rates depend on the crops as some, like mung bean, have shown significant increases in grain Zn concentration and yield at higher rates [55]. [56] also noted increased Zn uptake with different Zn-application methods, such as seed treatment and soil and foliar applications [57]. also found increased Zn uptake in common beans with Zn-amino acid chelates and foliar Zn applications. Despite improved Zn uptake, our results indicate that the uptake was considerably low, ranging between 0.016 and 0.198 kg ha^−1^ in finger millet and 0.017 and 0.163 kg ha^−1^ in soybean. This is consistent with past studies reporting slow Zn translocation in plant tissue due to an antagonistic relationship with P [49,58,59]. Improved grain Zn concentration and uptake might be attributed to the involvement of Zn in nutrient metabolism, which enhances nutrient absorption and overall crop performance [60].

### 4.4 Economic benefit of investing Zn fertilizer for finger millet and soybean grain production

The economic benefit of the fertilizer use was affected by fertilizer cost, grain prices, and how finger millet and soybean respond to the fertilizer application, as shown in Table 7, Table 8 Our study reveals that small-scale farmers can benefit from adding Zn fertilizer inputs if the fertilizers are affordable for finger millet and soybean production. It is widely accepted that a VCR of two and above is the typical and reliable profitable benchmark for local farmers to invest on a particular fertilizer [61]. , Our results largely show VCR values > 1 with Zn application. Specifically, growing finger millet, U15 variety (VCR = 2.05) in the long rains and SEC915 (VCR = 2.30) in the short rains with Zn application up to 3 kg ha^−1^ generated greater VCR values relative to the costs incurred on fertilizer during production. For soybean only, SB19 grown in Bungoma during long rains with 1.5 kg ha^−1^ Zn fertilizer recorded VCR of ≥2. These results are consistent with [62], who reported low VCR values in SSA compared to other regions worldwide. The low VCR values (≤2) reported in SSA are attributed to the socio-economic constraints and other constraints such as the high cost of fertilizer and less accessibility owing to poor development of markets and road infrastructure [63]. Therefore, we suggest offering appropriate fertilizer management strategies and knowledge to farmers to improve finger millet and soybean yield from Zn fertilizer inputs under Zn-poor soils in SSA.

## 5 Conclusion

The study revealed that Zn fertilizer use significantly enhances the grain yield, AE_Zn_, Zn concentration, and Zn uptake in both finger millet and soybean in western Kenya. The highest Zn application rate (3 kg ha⁻^1^) resulted in the largest grain yields, Zn Concentrations, and uptake across all varieties, seasons, and sites. However, the lower rate (1.5 kg ha^−1^ Zn) resulted in the highest Zn use efficiency, suggesting an optimal balance yield and efficiency. Value cost analysis revealed that Zn fertilizer use, particularly at 1.5 kg ha^−1^, offers significant economic benefits, making it a viable option for small-scale farmers. Varietal and site-specific responses highlighted the importance of selecting responsive crop varieties and tailoring fertilizer use strategies to local conditions for optimal results.

These findings underscore the critical role of Zn fertilizer application in improving crop productivity and nutritional quality in Zn-deficient soils, addressing both agricultural and public health challenges. We recommend a Zn application of 1.5 kg ha^−1^ alongside integrated soil fertility management practices for the best economic return and agronomic outcomes. By enhancing grain yield and Zn content in food crops such as finger millet and soybean, Zn fertilizer use can improve food and nutritional security in the regions. The insights gained from this research are valuable for farmers, agronomists, and policymakers focused on improving food security and nutrition through improved crop management.

## Data availability statement

The data generated for this study will be made available on request.

## CRediT authorship contribution statement

**Victor Ouma Oluoch:** Writing – review & editing, Writing – original draft, Visualization, Methodology, Formal analysis, Data curation, Conceptualization. **Abigael N. Otinga:** Writing – review & editing, Visualization, Validation, Supervision, Resources, Project administration, Methodology, Investigation, Funding acquisition, Formal analysis, Conceptualization. **Ruth Njoroge:** Writing – review & editing, Visualization, Validation, Supervision, Resources, Project administration, Methodology, Investigation, Formal analysis, Conceptualization.

## Declaration of competing interest

The authors declare that they have no known competing financial interests or personal relationships that could have appeared to influence the work reported in this paper.

## Figures and Tables

**Table 1:** Initial soil characterization for the study sites

Soil Parameters	Bungoma	Siaya
pH (water)	5.34	5.68
Total N (%)	0.23	0.29
Available P (mg kg^−1^)	6.48	5.86
Exchangeable K (mg kg^−1^)	69.60	321.43
Exchangeable Zn (mg kg^−1^)	7.06	7.57
Organic C (%)	1.46	1.63
**Texture**		
Sand (%)	64.95	44.22
Silt (%)	13.45	17.26
Clay (%)	21.63	38.52
**Textural class**	**sandy clay loam**	**clay loam**

Analysis was done following the standard procedure described in Okalebo et al. (2002).

**Table 2:** Treatment structure and description

		Treatment	Nutrient application (kg ha^−1^)				Nutrient Source
Crop	Variety	Codes	N	P	K	Zn	
Finger millet	U15	Control	0	0	0	0	no fertilizer
	U15	Zn1	100	30	50	0	Urea, TSP, MOP
	U15	Zn2	100	30	50	1.5	Urea, TSP, MOP, ZnO
	U15	Zn3	100	30	50	3	Urea, TSP, MOP, ZnO
	SEC915	Control	0	0	0	0	no fertilizer
	SEC915	Zn1	100	30	50	0	Urea, TSP, MOP
	SEC915	Zn2	100	30	50	1.5	Urea, TSP, MOP, ZnO
	SEC915	Zn3	100	30	50	3	Urea, TSP, MOP, ZnO
Soybean	SB19	Control	–	0	0	0	no fertilizer
	SB19	Zn1	–	30	45	0	TSP, MOP
	SB19	Zn2	–	30	45	1.5	TSP, MOP, ZnO
	SB19	Zn3	–	30	45	3	TSP, MOP, ZnO
	SB134	Control	–	0	0	0	no fertilizer
	SB134	Zn1	–	30	45	0	TSP, MOP
	SB134	Zn2	–	30	45	1.5	TSP, MOP, ZnO
	SB134	Zn3	–	30	45	3	TSP, MOP, ZnO

TSP = Triple superphosphate, MOP = Murate of Potash, ZnO = Zinc oxide, Control = no fertilizer, Zn1 = without Zn, Zn2= Zn (1.5 kg ha^−1^), Zn3 = Zn (3 kg ha^−1^), U15 = improved red seeded finger millet, SEC915 = improved brown seeded finger millet, SB19 = improved small-seeded soybean, SB134 = improved big seeded soybean.

**Table 3:**
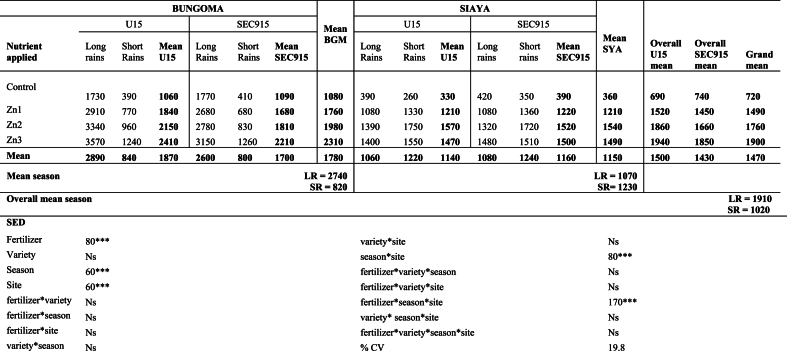
Finger millet grain yield (kg ha^−1^) as influenced by Zn application in Bungoma and Siaya during the 2019 LRs and SRs seasons

BGM-Bungoma, SYA-Siaya, LR – long rains, SR-short rains, SED – Standard error of the difference of means *** = significant at p ≤ 0.001, Ns = not significant control = no fertilizer, Zn1 = without Zn, Zn2= Zn (1.5 kg ha^−1^), Zn3 = Zn (3 kg ha^−1^), U15 = improved red seeded finger millet, and SEC915 = improved brown seeded finger millet.

**Table 4:**
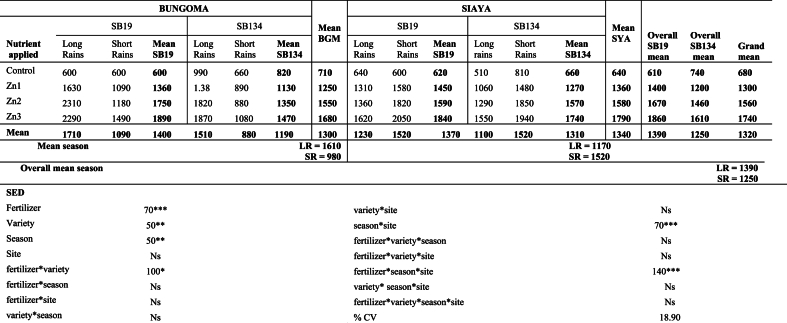
Soybean grain yield (kg ha^−1^) as influenced by Zn application in Bungoma and Siaya during the 2019 LRs and SRs seasons

BGM-Bungoma, SYA-Siaya, LR-long rains, SR-short rains, SED – Standard error of the difference of means *, ** and *** = significant at p ≤ 0.05, Ns = not significant, p ≤ 0.01 and p ≤ 0.001 respectively, control = no fertilizer, PKZn1 = without Zn, Zn2= Zn (1.5 kg ha^−1^), Zn3 = Zn (3 kg ha^−1^), SB19 = improved small-seeded soybean, and SB134 = improved big seeded soybean.

**Table 5:**
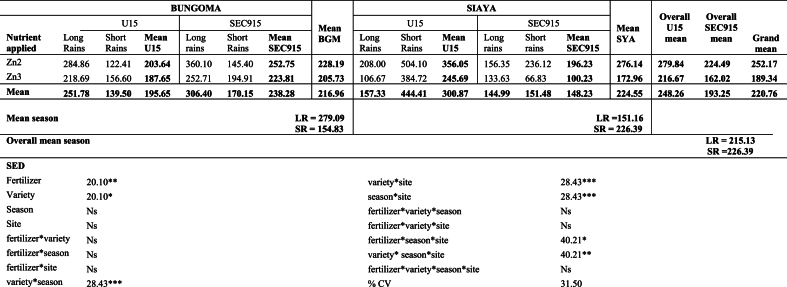
Agronomic efficiency of Zn in finger millet (kg kg^−1^) as influenced by Zn application in Bungoma and Siaya during the 2019 LRs and SRs seasons

BGM-Bungoma, SYA-Siaya, LR-long rains, SR-short rains SED – Standard error of the difference of means *,** and *** = significant at p ≤ 0.05, Ns = not significant, p ≤ 0.01 and p ≤ 0.001 respectively, control = no fertilizer, Zn1 = without Zn, Zn2= Zn (1.5 kg ha^−1^), Zn3 = Zn (3 kg ha^−1^), U15 = improved red seeded finger millet, and SEC915 = improved brown seeded finger millet.

**Table 6:**
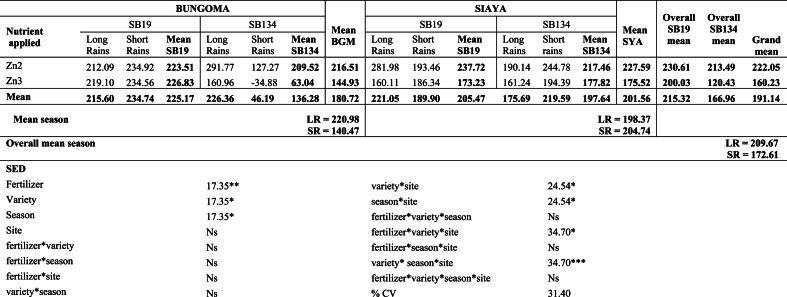
Agronomic efficiency of Zn in soybean (kg kg^−1^) as influenced by Zn application in Bungoma and Siaya during the 2019 LRs and SRs seasons

BGM-Bungoma, SYA-Siaya, LR-long rains, SR-short rains, SED – Standard error of the difference of means *, ** and *** = significant at p ≤ 0.05, Ns = not significant, p ≤ 0.01 and p ≤ 0.001 respectively, control = no fertilizer, Zn1 = without Zn, Zn2= Zn (1.5 kg ha^−1^), Zn3 = Zn (3 kg ha^−1^), SB19 = improved small-seeded soybean, and SB134 = improved big seeded soybean.

**Table 7:** VCR for finger millet

	Bungoma							Siaya						
LR	U15			SEC915				U15			SEC915			
Nutrient applied	Yield return ($ ha^−1^)	Fertilizer cost ($ ha^−1^)	**VCR**	Yield return ($ ha^−1^)	Fertilizer cost ($ ha^−1^)	**VCR**	VCR means BGM	Yield return	fertilizer cost ($ ha^−1^)	**VCR**	Yield return ($ ha^−1^)	fertilizer cost ($ ha^−1^)	**VCR**	VCR means SYA
Control	–	–	**-**	–	–	**-**	–	–	–	**-**	–	–	**-**	–
Zn1	1770.44	310.74	**5.70**	1355.95	310.74	**4.36**	5.03	1026.07	310.74	**3.30**	990.24	310.74	**3.19**	3.24
Zn2	2411.37	333.18	**7.24**	1513.98	333.18	**4.54**	5.89	1494.08	333.18	**4.48**	1342.02	333.18	**4.03**	4.26
Zn3	2754.55	355.50	**7.75**	2060.08	355.50	**5.79**	6.77	1506.06	355.50	**4.24**	1591.56	355.50	**4.48**	4.36
**Mean**	**2312.12**	**333.14**	**6.90**	**1643.34**	**333.14**	**4.90**	**5.90**	**1342.07**	**333.14**	**4.01**	**1307.94**	**333.14**	**3.90**	**3.95**
**SR**														
Control	–	–	**-**	–	–	**-**	–	–	–	**-**	–	–	**-**	–
Zn1	582.35	310.74	**1.87**	399.38	310.74	**1.29**	1.58	1611.74	310.74	**5.19**	1512.81	310.74	**4.87**	5.03
Zn2	857.77	333.18	**2.57**	633.66	333.18	**1.90**	2.24	2239.13	333.18	**6.72**	2044.07	333.18	**6.14**	6.43
Zn3	1287.06	355.50	**3.62**	1276.45	355.50	**3.59**	3.61	1935.63	355.50	**5.44**	1737.52	355.50	**4.89**	5.17
**Mean**	**909.06**	**333.14**	**2.69**	**769.83**	**333.14**	**2.26**	**2.48**	**1928.83**	**333.14**	**5.78**	**1764.80**	**333.14**	**5.30**	**5.54**
**VCR SED**														
Fertilizer		0.029*		fertilizer*site			Ns		fertilizer*season*site				Ns	
Variety		Ns		variety*season			Ns		variety*season*site				Ns	
season		0.023*		variety*site			Ns		fertilizer*variety*season*site				Ns	
Site		Ns		season*site <0.001***					CV%				37	
fertilizer*variety		Ns		fertilizer*variety*season			Ns							
fertilizer*season		Ns		fertilizer*variety*site			Ns							

BGM-Bungoma, SYA-Siaya, LR-long rains, SR-short rains SED – Standard error of the difference of means, VCR = value cost ratio for finger millet in both sites and seasons, $ ha^−1^ = US dollar per hectare, * and *** = significant at p ≤ 0.05, and p ≤ 0.001 respectively, Ns = not significant, control = no fertilizer, Zn1 = without Zn, Zn2= Zn (1.5 kg ha^−1^), Zn3 = Zn (3 kg ha^−1^), U15 = improved red seeded finger millet, and SEC915 = improved brown seeded finger millet.

**Table 8:** VCR for soybean

	Bungoma							Siaya						
LR	SB19			SB134				SB19			SB134			
Nutrient applied	yield return ($ ha^−1^)	Fertilizer cost ($ ha^−1^)	**VCR**	yield return ($ ha^−1^)	Fertilizer cost ($ ha^−1^)	**VCR**	VCR mean BGM	yield return ($ ha^−1^)	Fertilizer cost ($ ha^−1^)	**VCR**	yield return ($ ha^−1^)	fertilizer cost ($ ha^−1^)	**VCR**	VCR Mean SYA
Control	–	–	**-**	–	–	**-**	–	–	–	**-**	–	–	**-**	–
Zn1	1031.57	180.60	**5.71**	396.38	180.60	**2.19**	3.95	668.89	180.60	**3.70**	556.15	180.60	**3.08**	3.39
Zn2	1714.46	203.04	**8.44**	830.71	203.04	**4.09**	6.27	719.04	203.04	**3.54**	787.02	203.04	**3.88**	3.71
Zn3	1688.86	225.36	**7.49**	879.25	225.36	**3.90**	5.70	980.86	225.36	**4.35**	1039.87	225.36	**4.61**	4.48
**Mean**	**1478.30**	**203.00**	**7.21**	**702.11**	**203.00**	**3.39**	**5.31**	**789.60**	**203.00**	**3.86**	**794.35**	**203.00**	**3.86**	**3.86**
**SR**														
Control	–	–	**-**	–	–	–	–	–	–	–	–	–	–	–
Zn1	491.78	180.60	**2.72**	226.75	180.60	**1.26**	1.99	982.51	180.60	**5.44**	663.39	180.60	**3.67**	4.56
Zn2	580.92	203.04	**2.86**	223.81	203.04	**1.10**	1.98	1219.19	203.04	**6.00**	1030.56	203.04	**5.08**	5.54
Zn3	890.83	225.36	**3.95**	417.88	225.36	**1.85**	2.90	1452.32	225.36	**6.44**	1121.70	225.36	**4.98**	5.71
**Mean**	**654.51**	**203.00**	**3.18**	**289.48**	**203.00**	**1.40**	**2.29**	**1218.01**	**203.00**	**5.96**	**938.55**	**203.00**	**4.58**	**5.27**
**VCR SED**														
Fertilizer		0.038*		fertilizer*site			Ns		fertilizer*season*site				Ns	
Variety <0.001***				variety*season			Ns		variety*season*site				0.034*	
season		0.046*		variety*site			0.01**		fertilizer*variety*season*site				Ns	
Site		Ns		season*site <0.001***					CV%				39.6	
fertilizer*variety		Ns		fertilizer*variety*season			Ns							
fertilizer*season		Ns		fertilizer*variety*site			Ns							

BGM-Bungoma, SYA-Siaya, LR-long rains, SR-short rains, SED – Standard error of the difference of means, VCR = value cost ratio values for soybean in both sites and seasons, $ ha^−1^ = US dollar per hectare, *, ** and *** = significant at p ≤ 0.05,Ns = not significant, p ≤ 0.01 and p ≤ 0.001 respectively, control = no fertilizer, Zn1 = without Zn, Zn2= Zn (1.5 kg ha^−1^), Zn3 = Zn (3 kg ha^−1^), SB19 = improved small-seeded soybean, and SB134 = improved big seeded soybean.
